# Supplementary material for: Changes following the Onset of the COVID-19 Pandemic in the Burden of Hospitalization for Respiratory Syncytial Virus Acute Lower Respiratory Infection in Children under Two Years: A Retrospective Study from Croatia
Source: Viruses. 2022 Dec 9;14(12):2746. doi: 10.3390/v14122746 (PMC9785187; doi:10.3390/v14122746)
Supplement: Supplementary file 1 [file viruses-14-02746-s001.zip › viruses-2059513-supplementary.pdf]

Supplementary Material

Supplementary Table S1. Comparison between study subjects tested and not tested for RSV, and between study subjects testing positive and negative for RSV

	All-cause ALRI (N = 942)			ALRI tested for RSV (N =708)		
	**Tested for RSV (N =695)**	**Not tested for RSV (N =247)**	***P* value**	**Positive for RSV (N =282)**	**Negative for RSV (N =426)**	***P* value**
Sex, N (%)						
Male	397 (57.1)	148 (59.9)	0.445[a]	165 (58.5)	238 (55.9)	0.487[a]
Female	298 (42.9)	99 (40.1)		117 (41.5)	188 (44.1)	
Age group, N (%)						
0–<28d	72 (10.4)	10 (4.0)	<0.001[a]	31 (10.1)	41 (9.6)	<0.001[a]
28d–<3m	293 (42.2)	10 (4.0)		139 (49.3)	154 (36.2)	
3–<6m	162 (23.3)	31 (12.6)		68 (24.1)	94 (22.1)	
6–<12m	88 (12.7)	49 (19.8)		29 (10.3)	57 (13.4)	
12–<24m	80 (11.5)	117 (47.4)		15 (5.3)	80 (18.8)	
Gestational age, N (%)						
<28w	14 (20.1)	1 (0.4)	0.004[a]	2 (0.7)	12 (2.8)	0.156[a]
28–<32w	14 (20.1)	3 (1.2)		4 (1.4)	10 (2.3)	
32–<37w	79 (11.4)	12 (4.9)		35 (12.4)	44 (10.3)	
≥37w	588 (84.6)	231 (93.5)		241 (85.5)	360 (84.5)	
Birth weight, median (IQR)	3450 (3005,3780)	3430 (3095, 3840)	0.446[b]	3475 (3010, 3817.5)	3450 (3002.5, 3740)	0.362[b]
Birth height, median (IQR)	50 (49, 52)	51 (49, 52)	0.011[b]	50 (49, 52)	50 (49,52)	0.590[b]
Comorbidity, N (%)						
Any	74 (10.6)	16 (6.5)	0.055[a]	15 (5.3)	59 (13.8)	<0.001[a]
Chronic heart disease	35 (5.0)	6 (2.4)	0.084[a]	10 (3.5)	25 (5.9)	0.215[a]
Chronic pulmonary disease	26 (3.7)	4 (1.6)	0.103[c]	4 (1.4)	22 (5.2)	0.013[c]
Neuromuscular disease	3 (0.4)	0 (0.0)	0.571[c]	0 (0.0)	3 (0.7)	0.280[c]
Immunodeficiency	1 (0.1)	0 (0.0)	-	0 (0.0)	1 (0.2)	-
Down syndrome	9 (1.3)	6 (2.4)	0.221[a]	1 (0.4)	8 (1.9)	0.094[c]
Severity of ALRI, N (%)						
Severe	38 (5.5)	8 (3.2)	0.163[a]	13 (4.6)	27 (6.3)	0.330[a]
Very severe	23 (3.3)	5 (2.0)	0.307[a]	9 (3.2)	14 (3.3)	0.944[a]
Length of hospital stay, median (IQR)	5 (4, 6)	6 (4, 8)	<0.001[b]	6 (4,7)	6 (4,8)	0.063[b]

Note: ALRI, acute lower respiratory infection; RSV, respiratory syncytial virus.
a P-value calculated by Chi-square test
b P-value calculated by Mann-Whitney U test
c P-value calculated by Fisher's exact test

Supplementary Table S2. The association of testing for RSV with admission period relative to the COVID-19 pandemic, stratified by age group.

	Pre-COVID-19 period	During COVID-19 period	Odds Ratio (OR)
0-<6m			2.34 (1.30-4.21)
Non-tested for RSV	65 (16.25)	16 (7.66)	
Tested for RSV	335 (83.75)	193 (92.34)	
6-<12m			2.38 (0.98-5.79)
Non-tested for RSV	39 (41.94)	10 (23.26)	
Tested for RSV	54 (58.06)	33 (76.74)	
12-<24m			2.95 (1.50-5.79)
Non-tested for RSV	96 (66.67)	21 (40.38)	
Tested for RSV	48 (33.33)	31 (59.62)	
Overall odds ratio (M-H method)	-	-	2.53 (1.72-3.71)

Note: RSV, respiratory syncytial virus; m, months; OR, oodds ratio.

Supplementary Table S3. Multivariate logistic regression results of the risk factors for increased severity of all-cause ALRI hospitalizations by age group.

	0-<6 months		6-<12 months		12-<24 months	
Variables	Estimate	95% CI	Estimate	95% CI	Estimate	95% CI
Male	0.91	0.46-1.82	1.21	0.4-3.95	0.69	0.23-2.07
Preterm (<37wGA)	0.46	0.16-1.49	0.82	0.17-4.99	0.14	0.02-0.86
Low birth weight (<2500g)	0.86	0.23-3.06	1.26	0.19-6.51	0.12	0.01-0.98
Any comorbidity	5.45	2.18-12.88	2.34	0.49-9.65	16.9	3.79-80.78
Admitted after Mar 2020	2.48	1.28-4.87	0.43	0.09-1.52	3.98	1.35-12.3

Note: RSV, respiratory syncytial virus; CI, confidence interval; w, week; GA, gestational age.

Supplementary Table S4. Multivariate logistic regression results of the risk factors for increased severity of RSV-associated ALRI hospitalizations in 0-<6 months.

	0-<6 months	
**Variables**	**Estimate**	**95% CI**
Male	0.99	0.35-2.95
Preterm (<37wGA)	0.68	0.14-4.36
Low birth weight (<2500g)	2.87	0.47-16.06
Any comorbidity	0.93	0.04-7.07
Admitted after Mar 2020	7.65	2.54-28.69

Note: ALRI, acute lower respiratory infection; RSV, respiratory syncytial virus; CI, confidence interval; w, week; GA, gestational age. Analysis was not possible for the other two age groups (i.e. 6–<12 months and 12–<24 months) due to sparse data.

Supplementary Table S5. Multivariate logistic regression results of the risk factors for increased severity of all-cause and RSV-associated ALRI hospitalizations, with the number of comorbidities replacing presence of any comorbidities as the independent variable.

	Odds ratio for all-cause ALRI		Odds ratio for RSV-associated ALRI	
**Variables**	**Estimate**	**95% CI**	**Estimate**	**95% CI**
Male	0.93	0.56-1.55	0.71	0.28-1.81
Age				
0–<6m	1	reference	1	Reference
6–<12m	1.41	0.70-2.69	2.38	0.61-7.74
12–<24m	1.57	0.84-2.82	0.89	0.05-5.31
Preterm (<37wGA)	0.47	0.22-1.11	1.07	0.25-5.93
Low birth weight (<2500g)	0.76	0.29-1.89	2.05	0.38-9.54
Any one additional comorbidity	3.27	2.05-5.21	0.58	0.03-3.24
Admitted after Mar 2020	1.91	1.16-3.15	5.02	2.00-13.86

Note: ALRI, acute lower respiratory infection; RSV, respiratory syncytial virus; CI, confidence interval; m, months; w, week; GA, gestational age.

Supplementary Table S6. Multivariate logistic regression results of the risk factors for increased severity of all-cause and RSV-associated ALRI hospitalizations, with individual comorbidities as separate independent variables.

	Odds ratio for all-cause ALRI		Odds ratio for RSV-associated ALRI	
**Variables**	**Estimate**	**95% CI**	**Estimate**	**95% CI**
Male	0.94	0.57-1.56	0.76	0.30-1.95
Age				
0–<6m	1	reference	1	Reference
6–<12m	1.34	0.65-2.59	3.02	0.76-10.19
12–<24m	1. 53	0.82-2.77	1.01	0.05-6.08
Preterm (<37wGA)	0.50	0.22-1.21	1.33	0.28-8.60
Low birth weight (<2500g)	0.65	0.23-1.72	1.67	0.27-8.29
Chronic heart disease	1.81	0.68-4.36	NE	NE-NE
Chronic pulmonary disease	6.10	2.19-17.20	6.19	0.19-12.86
Neuromuscular disease	8.10	0.35-93.46	NE	NE-NE
Imunodeficiency	NE	NE-NE	NE	NE-NE
Down syndrome	4.44	1.22-14.24	NE	NE-NE
Admitted after Mar 2020	1.98	1.19-3.28	5.28	2.08-14.85

Note: ALRI, acute lower respiratory infection; RSV, respiratory syncytial virus; CI, confidence interval; m, months; w, week; GA, gestational age; NE, not estimated due to sparse data.

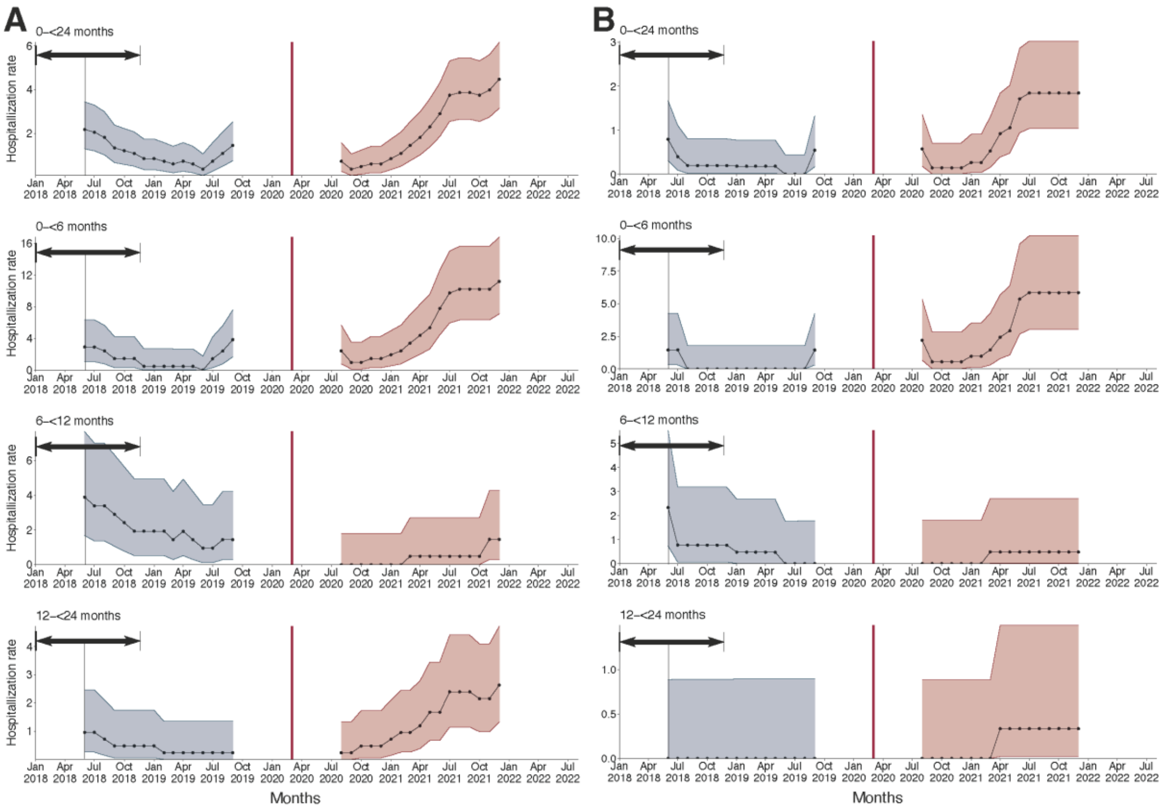

Supplementary Figure S1. Change in 12-month hospitalization rates of all-cause and RSV-associated ALRI by age group, before and after the onset of the COVID-19 pandemic. (**A**) 12-month all-cause ALRI moving average hospitalization rate. (**B**) 12-month RSV-associated ALRI moving average hospitalization rate. Red vertical line: lockdown in Croatia on 16 Mar 2020.
